# Accessible continued professional development for maternal mental health

**DOI:** 10.4102/phcfm.v11i1.1902

**Published:** 2019-01-31

**Authors:** Sally Field, Zulfa Abrahams, David L. Woods, Roseanne Turner, Michael N. Onah, Doreen K. Kaura, Simone Honikman

**Affiliations:** 1 Department of Psychiatry and Mental Health, University of Cape Town, South Africa; 2 School of Child and Adolescent Health, University of Cape Town, South Africa; 3 Faculty of Medicine and Health Sciences, Stellenbosch University, South Africa; 4 School of Public Health and Health Systems, University of Waterloo, Canada; 5 Institute of Fiscal Studies and Democracy, University of Ottawa, Canada; 6 Department of Nursing and Midwifery, Stellenbosch University, South Africa

## Abstract

**Background:**

Changing global health and development trends have resulted in a need for continued professional development (CPD) within the health and development sectors. In low-resource settings, where the need for training and CPD may be highest, there are significant challenges for disseminating information and skills. There is a need to improve mental health literacy and reduce levels of stigma about maternal mental illness. The Bettercare series of distance learning books provides a peer-based format for CPD. We aimed to evaluate the Bettercare *Maternal Mental Health* book as a format for CPD.

**Aim:**

The aim of this study was to determine whether the Bettercare *Maternal Mental Health* book significantly improves knowledge and decreases stigma around mental health for care providers from the health and social development sectors.

**Setting:**

One hundred and forty-one participants (social workers, nursing students and health professionals) were provided with the Bettercare *Maternal Mental Health* book to study.

**Methods:**

Before and after studying the book, the same multiple-choice knowledge test and the Mental Illness Clinicians’ Attitude Scale were used to assess cognitive knowledge and mental health stigma, respectively.

**Results:**

Participants’ knowledge showed a statistically significant (*p* < 0.001) improvement between the pre- and post-test results, for all six chapters of the book. However, participants’ attitudes towards mental illness did not show a statistically significant change between the pre- and post-test results.

**Conclusion:**

We found that this method of learning elicited significant improvement in mental health knowledge for care providers. Continued professional development policy planners and curriculum developers may be interested in these findings.

## Introduction

Changing global health and development trends have resulted in a need for continued professional development (CPD) within the health and development sectors. Initial training no longer equips care providers adequately to address the changes that occur during their professional lifetime.^1^

Rapid medical and technological advancements challenge care providers to keep up with new trends, while the regulation of professional bodies has influenced the need for standardised practice with set curricula and thus the need for CPD in order for professionals to update their knowledge.^2^ Changing demographic patterns of disease have significant impacts on health delivery systems,^1^ resulting in the need for care providers to keep abreast with new evidence and relevant skills in order to be able to respond appropriately.

There have been several global initiatives to address the issue of CPD. More traditional forms of CPD include conferences and workshops,^3^ with more recent initiatives including the use of social media platforms.^4^ However, numerous challenges have been identified. These include juggling workload and time for study, inflexible education systems with scheduled lectures that do not take the work shift timetable into account and a gap between theory and practice.^1^ Further, the uptake and impact of professional development is influenced by lack of relief cover, inability to take paid study leave and an unsupportive learning culture,^5^ together with other constraints such as a lack of funding to support training initiatives and experienced trainers.

In low-resource settings, where the need for training and CPD may be highest, there are even greater obstacles to disseminating information and skills. Traditional methods of continued training usually occur off-site, in urban areas, at tertiary training institutions or central teaching hospitals. For those living remotely, the expenses in terms of time, accommodation and transport are often prohibitive.^6^ While the use of social media and online platforms for CPD may enable care providers to remain at their place of employment while continuing their education, these options are currently inaccessible for most South Africans outside of the major metropolitan cities because of expensive data costs and lack of reliable free Wi-Fi.

In South Africa, 75% of people living with a mental disorder do not receive the care that they need.^7^ Common mental disorders (CMD) such as depression and anxiety are highly prevalent in low-resource settings, particularly during the perinatal period. In South Africa, perinatal CMDs are as high as 47% in rural settings^8^ and more than 20% in urban settings.^9,10^ Research has indicated the need for strengthening health systems in order to address the gap in mental healthcare delivery. This includes the need for further training of non-specialist care providers (health and social workers) at different levels.^11^ However, studies from South Africa report negative attitudes of care providers towards those with mental illness.^12,13,14^ Mavundla^12^ reported that 90% of nurses working in a general hospital in Durban had negative attitudes towards their mentally ill patients. As institutional stigma decreases the uptake of services by those with mental illness,^15^ there is thus a need to improve the mental health literacy of care providers as well as to reduce levels of stigma about mental illness.

The book *Maternal Mental Health: A Guide for Health and Social Workers* is one of many in the Bettercare series of distance learning books and was written by the authors of this article (SF, DW, SH). The Bettercare books provide relevant, affordable and up-to-date learning materials for care providers in under-resourced settings. The series covers a range of health topics (http://bettercare.co.za/). Each course book follows the same format: learning objectives are clearly stated at beginning of each chapter, chapter content is presented in a question-and-answer format and contains case studies that apply the chapter content to practical, real-world settings. The case studies include prompt questions for considered assessment and management options as well as model answers. Each chapter has a multiple-choice quiz consisting of 20 questions to self-assess knowledge and understanding before and after studying the chapter. The correct answers are provided at the back of the book. The first edition of the *Maternal Mental Health* book is divided into six chapters: ‘Introduction to maternal mental health’, ‘Identifying maternal mental illness’, ‘Making referrals for maternal mental illness’, ‘How to help mothers with mental health problems’, ‘Special medical issues in maternal mental health’ and ‘Special social issues in maternal mental health’. The book also has a resource section with details on screening tools and accessing help for pregnancy termination, adoption, maintenance and protection orders, child support grants, as well as helplines and organisations that can assist with counselling services.

While a need to improve the mental health knowledge, understanding and attitudes of care providers has been identified, there is little agreement on strategies for training or for the evaluation of training efforts. An African systematic review on training in mental health identified key gaps for research that develops and tests innovative educational strategies, especially those that support integration of mental health systems.^16^ We aimed to evaluate the changes in mental health knowledge and stigma in non-specialist health and social workers using the Bettercare *Maternal Mental Health* book as an innovative educational strategy for distance learning CPD.

## Methods

### Study design

This study used a pre-post-study design.

### Participants

Participants for this study were recruited in one of three ways. Firstly, social workers who enrolled for training in maternal mental health through a Western Cape Department of Social Development CPD programme were requested to participate in the study prior to attending a workshop – there were 59 participants recruited in this manner, with no refusals to participate. Secondly, basic and advanced midwifery students from the University of Stellenbosch were asked to participate in the study as part of their coursework – there were 55 participants from this group, with no one declining to participate. The remaining 27 participants were health or social worker students and professionals who had accessed the *Maternal Mental Health* book online and volunteered to participate in the study.

### Procedure

All participants were provided with a copy of Bettercare *Maternal Mental Health*, free of charge. The study process was clearly explained, either in person by a facilitator or online, in written form. Participants were encouraged to form small study groups, ideally of about five people, and to work their way through the course book, chapter by chapter, discussing the concepts and case studies. Participants were required to keep an attendance register that documented attendance, dates of group meetings and the chapters that were studied at each session. Individuals who participated in the study outside of the group format were not required to document their study times.

### Data collection

A self-administered questionnaire was used to collect socio-demographic information from participants at the beginning of the study. The Mental Illness Clinicians’ Attitude Scale (MICA-4) was used to assess participants’ stigma towards people with mental illness ^17^ before and after participating in the study of the *Maternal Mental Health* book. The MICA-4 version was designed to assess health and social care professionals’ attitudes, views and knowledge. It consists of 16 questions with a six-point Likert scale, which was completed as a self-administered survey. The lowest possible score on the scale is 16, and the highest 96. Ten of the items are reverse scored. A high overall score is an indication of a more negative (stigmatising) attitude. It has been used in other African settings^18^ and in South Africa with nurses in primary care clinics.^19^

Before participating in the self-study process, participants completed a multiple-choice test consisting of 75 questions. These were devised by the authors of the book and were peer-reviewed. The questions related to the content of the six chapters of the book. The same multiple-choice test was repeated after completion of the book, several weeks later. The results of the pretest indicated the participants’ knowledge of the subject matter before studying the material, while the post-test indicated their knowledge after studying it. The difference between the two results indicated the amount of knowledge gained in the interval.

To assess the perceived usefulness of the process, we asked participants about the difficulties they had experienced, as well as what aspects and sections of the content they found to be particularly helpful.

### Data analysis

Data analysis was performed using Stata/SE statistical software package version 14.0 (StataCorp, College Station, TX, Unites States of America [US]). Because the data were not normally distributed, the stigma and knowledge scores were described as medians with interquartile ranges and were compared using the Wilcoxon signed-rank test for paired samples. Participants’ comments on the difficulties they experienced and the most helpful areas of the book were analysed using simple thematic analysis.

### Ethical consideration

The study proposal was approved by the Research Ethics Committee of the Faculty of Health Sciences at the University of Cape Town (Ref. 474/2015) and from the University of Stellenbosch Health Research Ethics Committee (Ref. N15/10/105). Written informed consent was obtained from all participants prior to participation in the study.

## Results

The demographic characteristics of the 141 participants who completed the pre- and post-tests are shown in Table 1. The majority were aged between 26 and 35 years (43%) and were women (95%) working in South Africa (95%). Almost half the sample (47%) were social workers or social work assistants, while 37% were nurses. The remaining participants were registered professionals from the health or social development sectors and included doctors and a psychologist. More than half (58%) reported no previous mental health training. Not all demographic information was provided by all participants.

**TABLE 1:** Demographic characteristics of participants (*n* = 141).

Variable	Participants	
	*n*	%
**Age**		
< 25 years	20	12.2
26–35 years	60	42.6
36–45 years	31	22.0
> 46 years	30	21.3
**Gender**		
Men	7	4.9
Women	134	95.0
**Country of residence**		
South Africa	118	92.9
Other	10	5.7
**Employment status**		
Employed	118	83.7
Unemployed	9	7.1
**Location of residence**		
Rural	23	18.1
Urban	104	81.9
**Type of job**		
Nurse (professional/specialised/midwife/enrolled)	46	36.8
Social worker/social work assistant	59	47.2
Unspecified other	20	16.0
**Type of mental health training received**		
None	82	58.2
Degree	32	22.7
Workshop/seminar	11	7.8
Informal	13	9.2
**Study method used**		
In a group	102	72.3
Individually	39	27.7

Participants’ knowledge showed a statistically significant (*p* < 0.001) improvement (Table 2) between the pre- and post-test results, for all six chapters, irrespective of the method of recruitment. Only two participants failed to get a post-study score of 80% or above.

**TABLE 2:** Comparison of pre- and post-test scores for chapters in the maternal mental health course book (*n* = 141).

Chapter number	Chapter content and description	Number of questions	Pretest		Post-test		*p*-value*
			Median	IQR	Median	IQR	
1	*Introduction to maternal mental health*: outlines risk factors for poor maternal mental health and defines different types of maternal mental illnesses	19	15	13–16	19	17–19	< 0.001
2	*Identifying maternal mental illness*: outlines challenges for identification of maternal mental illness and provides screening tools for common mental disorders with explanations for their use	6	5	4–6	6	5–6	< 0.001
3	*Making referrals for maternal mental illness*: outlines how to refer, what resources may be available, explains why mothers may default on appointments	6	5	5–6	6	5–6	< 0.001
4	*How to help mothers with mental health problems*: outlines why pregnancy is particularly stressful and what assists or hinders care providers in alleviating these stressors	21	18	16–19	20	19–21	< 0.001
5	*Special medical issues in maternal mental health*: provides an understanding of HIV, substance misuse, suicide, postnatal psychosis and perinatal loss in relation to mental health issues	12	9	7–10	11	10–11	< 0.001
6	*Special social issues in maternal mental health*: provides an understanding of poverty, teen pregnancy, refugees, maternal and child abuse in relation to mental health issues	12	10	9–11	12	11–12	< 0.001

IQR, interquartile range.

* , Paired *t*-test.

Most subgroups (Table 3) showed statistically significant (*p* < 0.001) improvement in knowledge between the pre- and post-test. The exception was those who had received previous mental health training at a workshop or seminar (*p* = 0.111). The poorest median knowledge scores at baseline were for those who were 36–45 years old (57/75), men (57/75) and those who completed the programme on their own (57/75). Subgroups with the highest median knowledge scores at the post-test included men (73/75) and those who had received previous training at a workshop or seminar (74/75).

**TABLE 3:** Comparison of pre- and post-test knowledge scores (maximum possible score = 75).

Variable	Pretest score		Post-test score		*p*-value*
	Median	IQR	Median	IQR	
**Age**					
< 25 years	65	63–68	72	68–74	0.005
26–35 years	60	56–65	72	70–73	< 0.001
36–45 years	57	52–66	72	67–74	< 0.001
> 46 years	61	53–64	70	67–73	< 0.001
**Gender**					
Men	57	55–65	73	64–74	0.013
Women	61	55–65	71	69–73	< 0.001
**Country of employment**					
South Africa	60	55–65	72	68–73	< 0.001
Other	64	56–69	71	69–73	0.009
**Location of employment**					
Rural	63	56–66	71	67–73	< 0.001
Urban	61	55–65	72	68–73	< 0.001
**Type of job**					
Nurse (professional/specialised/ midwife/enrolled)	59	53–62	71	67–73	< 0.001
Social worker/social worker assistant	63	57–66	72	70–73	< 0.001
Unspecified other	65	61–69	71	65–74	0.034
**Type of mental health training received**					
None	58	53–63	72	69–73	< 0.001
Degree	64	60–67	71	68–73	< 0.001
Workshop/seminar	65	58–66	74	64–75	0.111
Informal	61	59–65	72	67–73	0.018
**Study method used**					
In a group	61	56–66	71	68–73	< 0.001
Individually	57	51–66	72	70–73	< 0.001

**Total score**	**61**	**55–66**	**71**	**69–73**	**< 0.001**

IQR, interquartile range.

* , Paired *t*-test.

Overall, the participants’ attitudes towards people with a mental illness did not show a statistically significant change between the pre- and post-test results (*p* = 0.486) (Table 4). The median score was 38 for the pretest, which decreased to 37 at the post-test.

**TABLE 4:** Comparison of pre- and post-test stigma scores on the Mental Illness Clinicians’ Attitude scale (*n* = 136)†.

Variable	Pretest stigma score		Post-test stigma score		*p*-value*
	Median	IQR	Median	IQR	
**Age**					
< 25 years	40	37–45	36	28–48	0.179
26-35 years	37	32–42	40	30–43	0.528
36-45 years	39	34–44	38	34–40	0.955
> 46 years	37	29–43	35	28–42	0.358
**Gender**					
Men	42	32–47	37	28–43	0.028
Women	37	32–42	37	30–42	0.636
**Country of employment**					
South Africa	38	33–42	37	29–42	0.259
Other	37	27–45	37	33–47	0.533
**Location of employment**					
Rural	40	34–44	40	34–44	0.855
Urban	37	32–43	37	32–43	0.249
**Type of job**					
Nurse (professional/specialised/ midwife/enrolled)	37	33–40	37	28–41	0.582
Social worker/social worker assistant	38	31–42	37	30–43	0.502
Unspecified other	36	30–47	40	35–43	0.654
**Type of mental health training received**					
None	39	34–44	38	30–41	0.033
Degree	35	31–41	37	30–47	0.594
Workshop/seminar	32	26–37	37	32–43	0.169
Informal	37	32–39	40	27–47	0.916
**Study method used**					
In a group	38	32–42	38	30–43	0.553
Individually	38	33–45	37	27–42	0.700

**Overall score**	**38‡**	**32–43**	**37**	**30–42**	**0.486**

IQR, interquartile range.

* , Paired Wilcoxon signed-rank test.

† , Five participants had incomplete questionnaires and therefore needed to be excluded from this sample.

‡ , A high overall score indicates a more negative (stigmatising) attitude.

There was no significant difference in change of knowledge score (*p* = 0.083) or change in stigma score (*p* = 0.964) for those who studied individually compared to those who participated in group study sessions.

When asked about areas that were difficult to understand in the book, the majority of participants (78%) reported having no difficulties. With regard to the content of the *Maternal Mental Health* book, eight participants indicated that they found the scoring of one of the screening tools difficult to understand, with six indicating that the screening tools were particularly helpful. Two social worker participants reported some difficulty in understanding some of the medical terminology.

Participants reported that the book contained ‘well-structured chapters’, ‘short paragraphs that were easy to read’ and ‘manageable segments of information’ and that the book was ‘easy to understand’.

The most helpful reported areas of the book included the management of mentally ill patients (27%), the entire book (26%), case studies (10%) and the resource section (10%).

Further, 14% of the participants provided examples of how they could apply their knowledge in their personal or work lives. Many participants reported being better equipped to identify and treat women with a mental illness. Others commented on their increased understanding and feelings of empathy for pregnant women with mental illnesses.

## Discussion

This study evaluated the Bettercare *Maternal Mental Health* book as a distance learning format. We found that this method of learning elicited significant improvement in maternal mental health knowledge for care providers in the health and social development sectors but was not effective at changing participants’ attitudes towards mental illness. The majority of participants reported that the chapters were well structured and easy to understand.

In a review of over 100 trials concerning continued professional education activities, Oxman et al. concluded that dissemination-only strategies, such as conferences or mailing of unsolicited information, may assist with providing new knowledge but demonstrated little change in professional behaviour.^20^ Educational scholars have argued that more complex cognitive processes are needed for putting knowledge into practice. These would include knowledge comprehension, application, analysis, synthesis and evaluation.^3^

The Bettercare method of using questions and answers followed by case studies attempts to integrate some of these more complex processes into learning, moving beyond knowledge comprehension. Participants are encouraged to apply the knowledge content by working through a series of real-life case study scenarios, analysing the information and then evaluating their change in knowledge through before and after quizzes. Peer group discussions encourage participants to engage actively with the material and assist each other with assimilating new knowledge. It is therefore not surprising that participants’ knowledge improved significantly between the pre- and post-test. Feedback on the content of the *Maternal Mental Health* book further demonstrates participants’ perception of their improved ability to apply their improved knowledge of maternal mental health in their personal and work environments. These findings concur with previous studies by Woods and Theron that this format of decentralised learning increases cognitive knowledge.^21^ Theron demonstrated significant change in knowledge among midwives who studied the *Maternal Care* book^22^ and Woods demonstrated similar success in his study that showed improved cognitive knowledge of *Neonatal Care* by neonatal nurses in a district hospital and satellite clinic.^23^ These studies drew on participants from both rural and urban areas from all levels of the health system.

In a prospective controlled trial, Theron found that the change in cognitive knowledge about maternal care also resulted in a change in participants’ attitudes towards their work.^24^ The midwives in his study reported that improved cognitive knowledge and ability to apply this knowledge was also personally experienced, resulting in an increase in job satisfaction and self-confidence. We did not measure these items. Rather, we looked at stigma around mental health as the important attitude being influenced by the process of engaging with the content of the book. We found that while participants’ attitudes towards mental illness did not change significantly, their stigma scores were reduced.

Greater mean scores on the MICA scale indicate higher levels of stigma towards mental health. The mean score in this study prior to the learning process was far lower than the mean scores for similar professionals and medical students who underwent training in identifying mental health disorders in China.^25,26^ In both these studies, the pre-and post-mean MICA scores ranged between 47 and 41. A Nigerian study used the MICA to assess university staff attitudes towards mental illness. Across all university faculties, the mean total scores ranged between 44 and 80, with faculty members from the psychiatry department scoring the lowest mean score of 44.^18^ The pretest MICA mean score for our study was 38 and the post-test had a non-significant lowered mean score of 37. This seems to suggest that even prior to completing the *Maternal Mental Health* book, our participants were less prejudiced about mental health issues than their contemporaries in China or Nigeria and had similar attitude scores to midwives in a study from Ireland (mean score of 36)^27^ and medical students at an English university (mean score of 34).^17^ We are unable to compare the scores to a South African study who surveyed primary care nurses, as a different scoring system was used. However, that study reported a ‘relatively positive attitude towards mental illness’ by primary care nurses.^19^

Studies reporting effective methods for reducing stigma seem to be limited, however, pilot interventions where participants have contact with those living with mental illness seem to be promising.^28^ Experiential learning, rather than paper-based study, may be necessary for changing negative attitudes towards mental illness. A review on interventions that have been successful in changing knowledge and attitudes towards mental illness indicates that personal experience and a focus on modelling skills and behaviour have been most effective.^29^ While the *Maternal Mental Health* book provides case studies to apply knowledge and model typical scenarios pertaining to maternal mental illness, this does not seem to be sufficient for significant changes in attitude. Some of the authors of this article, through the Perinatal Mental Health Project, have recently developed a short film to model empathic engagement (http://bit.ly/PMHP_ESS), aimed to supplement the book in providing visual demonstration of the skills.

An Australian study, looking at CPD in rural areas, found that professional isolation was one of the factors that stimulated the need for CPD activities.^30^ The peer group learning format encouraged by Bettercare addresses this. Participants are encouraged to learn in small groups, motivating each other and sharing knowledge in a collaborative way. The self-directed learning approach enables participants to study at flexible times and at their own pace. A formal tutor is not needed for this method of cooperative learning and peer tuition, although a facilitator is helpful.

While we did not find a significant difference in either change in knowledge or change in mental health stigma between those who studied individually compared to those who studied in a group setting, this could have been influenced by the method of recruiting participants into the study. More participants who studied the book as individuals self-selected to participate via online recruitment and thus may have been more motivated to engage with the content of the study.

## Limitations

The study is limited in that it has a relatively small sample size. While this study assessed knowledge and attitudes, it was not designed to examine how these were applied as skills and work performance. Further, we did not measure the long-term retention of knowledge.

Further, pre-and post-test design may be subject to a number of confounding variables such as maturation, history, test effect and the regression to the mean effect.^31^ These are noteworthy limitations when there is no comparison group against which to evaluate the findings.

We acknowledge that some of the authors of the *Maternal Mental Health* book are also the researchers in this study. While this may be a source of bias, effort was taken to limit this through use of other researchers for data collection and analysis, who were not directly linked to the development or the outcomes of the book.

Although pragmatic constraints in the recruitment process led to a large majority of urban participants, we anticipate the benefits of the *Maternal Mental Health* book would be equally felt for rural user groups. In particular, these populations may best be served by the online open access platform of the Bettercare series.

## Conclusion

Despite these limitations, the findings suggest that the Bettercare format could be an effective method for CPD and that the *Maternal Mental Health* book improves knowledge among different provider cadres who do not typically integrate mental healthcare in their routine practice. These findings may be of interest to those involved in the policy, planning and implementation of curricula for training and CPD for care providers in the health and social development sectors, particularly in regions where there is a high prevalence of maternal mental ill health and low levels of human resources to meet this need. Future research should seek to measure the application of mental health knowledge and skills in the workplace, as well as long-term retention of these. In particular, there is scope to research whether and how video training materials impact practice, particularly in the field of mental health.
